# Comparison of adverse maternal and perinatal outcomes between induction and expectant management among women with gestational diabetes mellitus at term pregnancy: a systematic review and meta-analysis

**DOI:** 10.1186/s12884-023-05779-z

**Published:** 2023-07-12

**Authors:** Rong Luo, Wendy Wen, Daniel J. Corsi, Deshayne B. Fell, Monica Taljaard, Shi Wu Wen, Mark C. Walker

**Affiliations:** 1 Clinical Epidemiology Program, Ottawa Hospital Research Institute, Ottawa, Canada; 2 School of Epidemiology and Public Health, University of Ottawa, Ottawa, Canada; 3 McLaughlin Centre for Population Health Risk Assessment, University of Ottawa, Ottawa, Canada; 4 Department of Obstetrics and Gynecology, University of Ottawa, Ottawa, Canada; 5 Children’s Hospital of Eastern Ontario Research Institute, Ottawa, Canada; 6 Department of Obstetrics, Gynecology & Newborn Care, The Ottawa Hospital, Ottawa, Canada; 7 International and Global Health Office, University of Ottawa, Ottawa, Canada

**Keywords:** Gestational diabetes mellitus (GDM), Term pregnancy, Induction, Expectant management, Caesarean section, Macrosomia

## Abstract

**Background:**

Induction at 38–40 weeks of gestation has been broadly suggested for women with gestational diabetes mellitus (GDM), yet its benefits and risks remain unclear. This study aimed to systematically review and meta-analyze existing evidence on the effect of induction at term gestation among women with GDM.

**Methods:**

We searched MEDLINE, EMBASE, Cochrane Libraries, and Web of Science from inception to June 2021. We included randomized controlled trials (RCTs) and observational studies comparing induction with expectant management among GDM term pregnancies. Primary outcomes included caesarean section (CS) and macrosomia. All screening and extraction were conducted independently and in duplicates. Meta-analyses with random-effects models were conducted to generate the pooled odds ratios (ORs) and 95% confidence intervals (CIs) using the Mantel-Haenszel method. Methodological quality was assessed independently by two reviewers using the Cochrane Risk of Bias Tool for RCTs and the Newcastle-Ottawa Scale for observational studies.

**Results:**

Of the 4,791 citations, 11 studies were included (3 RCTs and 8 observational studies). Compared to expectant management, GDM women with induction had a significantly lower odds for macrosomia (RCTs 0.49 [0.30–0.81]); observational studies 0.64 [0.54–0.77]), but not for CS (RCTs 0.95 [0.64–1.43]); observational studies 1.03 [0.79–1.34]). Induction was associated with a lower odds of severe perineal lacerations in observational studies (0.59 [0.39–0.88]). No significant difference was observed for other maternal or neonatal morbidities, or perinatal mortality between groups.

**Conclusions:**

For GDM women, induction may reduce the risk of macrosomia and severe perineal lacerations compared to expectant management. Further rigorous studies with large sample sizes are warranted to better inform clinical implications.

**Supplementary Information:**

The online version contains supplementary material available at 10.1186/s12884-023-05779-z.

## Background

Gestational diabetes mellitus (GDM), characterized as any carbohydrate intolerance with first detection during the late second trimester of pregnancy [1], has been considered one of the most common pregnancy disorders. Globally, GDM affects 7–10% of all pregnancies, depending on the screening and diagnostic criteria and population profile [2]. Although GDM mostly resolves after pregnancy, it imposes an increased risk of adverse outcomes in the short- and long-term, including gestational hypertensive disorders, macrosomia, postpartum type 2 diabetes and cardiovascular disorders for the mother and their offspring [3–5].

Induction of labour (IOL) has been increasing steadily and approximately 25% of labours are induced in high-income countries [6]. An increase in induction at term without clinically-accepted indications—termed “elective induction”—appears to be an important contributor to the overall upward trend [7]. Currently, IOL has been broadly suggested for GDM term pregnancies from different professional societies and institutions to improve pregnancy outcomes, despite inconsistent recommendations for timing of induction [8–11]. In Canada, the Society of Obstetricians and Gynaecologists of Canada (SOGC) and Diabetes Canada (DC) recommend offering IOL between 38 and 40 weeks for GDM pregnancies [8, 9]. While in the UK, the National Institute for Health and Care Excellence (NICE) recommends IOL by the 40th week [10], and in the US, the American College of Obstetricians and Gynecologists (ACOG) recommends induction between 39 and 40 weeks for well-controlled GDM pregnancies [11]. However, the majority of these recommendations were reached by expert consensus and few were supported by high-quality studies.

There is a paucity of systematic reviews of studies on induction in GDM women, and thus the benefits and risks of induction remain unclear. Previous studies assessing the effect of IOL yielded inconsistent results due to variations in population profile, diagnostic criteria of GDM, comparators and other methodological issues [12–14]. The only three systematic reviews published to date comparing induction versus expectant management in GDM term pregnancies also reported conflicting results, without firm conclusions [15–17]. The inconsistencies are most likely attributed to the differences in data sources and search strategy, inclusion and exclusion criteria, small number of studies and high heterogeneity, and low data quality.

To address this knowledge gap, our systematic review and meta-analysis was conducted to update the evidence pertinent to the benefits and risks of induction versus expectant management for women with GDM, and to explore the potential sources of heterogeneity underlying the inconsistent findings.

## Methods

This systematic review was performed and reported following the Preferred Reporting Items for Systematic Reviews and Meta-analyses guidelines (PRISMA 2020 statement) [18]. This study had no patient or public involvement and data were collected from existing literature, therefore, no ethical approval was required. The study protocol was registered on PROSPERO before starting the review (CRD#42021256268) [19].

### Searching strategy and selection criteria

We undertook a comprehensive literature search using electronic and manual searching. Four electronic databases, Ovid-MEDLINE (1946 – 2021 June), Ovid-EMBASE (1947 – 2021 June), Cochrane Libraries (inception − 2021 June), and Web of Science (inception − 2021 June), were systematically searched. An initial search strategy was developed in MEDLINE using Medical Subject Headings (MeSH) terms and text words related to the population and exposures of interest in consultation with a professional medical librarian and adapted for the other databases (Appendix S1). The reference lists of all identified articles were examined for potentially additional references. We also searched the WHO international Clinical Trials Registry Platform (ICTRP) and ClinicalTrials.gov to identify any ongoing or unpublished trials.

Criteria to identify eligible studies for the current review were guided by the PICOS (Population-Intervention-Comparators-Outcomes-Study design) framework. Studies were considered eligible if: [1] using experimental [i.e. randomized clinical trial (RCT)] or comparative observational (i.e. prospective or retrospective cohort, case-control) study designs; [2] the study comprised women who had a singleton term pregnancy (≥ 37 weeks) and were diagnosed with GDM in the index pregnancy; [3] they compared induction with expectant management; and [4] they addressed any of the maternal and neonatal outcomes. The primary outcomes comprised: CS and macrosomia (birthweight ≥ 4,000 g, or as defined by study authors), while secondary outcomes included: instrumental vaginal delivery (by forceps or vacuum), severe perineal lacerations (third- and fourth-degree perineal tears), intensive care unit (ICU) admission, large-for-gestational-age (LGA) neonates (birthweight higher than the 90th percentile for the same gestational age and sex, or as defined by study authors), shoulder dystocia (delivery requiring additional obstetric manoeuvres to release infant’s shoulder after failure of gentle downward traction), neonatal intensive care unit (NICU) admission, 5-min Apgar score < 7, neonatal acidemia (umbilical cord artery PH < 7 and/or a base deficit > 12 mmol/l, or as defined by study authors), and perinatal mortality (intrauterine fetal death and neonatal death within 28 days following livebirth, or as defined by study authors). For studies involving diabetic pregnant women, if there was no distinction between diabetes types, or less than 90% of subjects had GDM but without subgroup analysis by diabetes type, they were ineligible. Studies published as conference abstracts were deemed eligible if there were sufficient information for data extraction and quality assessment. Narrative reviews, protocols, commentary and correspondence were excluded. Finally, we excluded studies not published in English or if they used a clinically improper comparison including using inappropriate intervention or comparator (e.g. spontaneous vaginal delivery, induction at a later gestational week).

We imported all search records into Mendeley citation manager to remove any duplicates; remaining records were then imported into Covidence for screening. All screenings (title/abstract and full-text reviewing) were conducted independently and in duplicates by two reviewers (R.L. and W.W.). Any disagreement was resolved by discussion with a third reviewer (S.W.W.).

### Data extraction and risk of bias assessment

Two reviewers (R.L. and W.W.) independently extracted the following data into a standard and prespecified form on Microsoft Excel spreadsheet in a duplicate manner: author and publication year, journal, country, study period, study design, population demographics, GDM diagnostic criteria, GDM treatment/subtype, inclusion/exclusion criteria, sample size, definition and ascertainment of interventions and outcomes, number of events, crude and/or adjusted relative effect estimates (relative risk [RR] or odds ratio [OR]) with 95% confidence intervals (CIs) and adjusted variables. Where available, data were also abstracted on induction methods and timing. When the impact of induction was assessed by multiple gestational weeks, only the results of induction at 38 weeks were extracted. The original authors were contacted for further details and clarity when needed.

The methodological quality of each study was independently assessed by two reviewers (R.L. and W.W.) according to the study design. Specifically, RCTs were assessed using the Cochrane Risk of Bias Tool [20] to classify RCTs as having a low, unclear or high risk of bias. The observational studies were assessed using the Newcastle-Ottawa Scale (NOS) [21] to classify the studies as being of poor, fair or good quality. Additionally, the overall quality of evidence for all outcomes across studies was evaluated using the GRADE (Grading of Recommendations Assessment, Development, and Evaluation) approach [22] and rated as very low, low, moderate or high in terms of risk of bias, inconsistency, imprecision and indirectness by the GRADEPro Guideline Development Tool (GDT).

### Data synthesis and analysis

Data extracted from included studies were compiled into 2 × 2 tables which were consisted of the numbers of events and non-events in intervention and comparison groups. All analyses were completed in RevMan (version 5.4). Original studies with similar outcomes were pooled together and the 2 × 2 tables were used to calculate the crude ORs. For studies without explicit event data, we estimated the absolute numbers according to the reported percentages. If no event was recorded in one group, we applied a fixed correction using the default value of 0.5 to each cell of the 2 × 2 tables to avoid division by zero; if no events were presented in both groups, we excluded those studies from pooled analysis for specific outcomes.

Similarity across studies was assessed in terms of clinical heterogeneity (e.g., clinical characteristics), methodological heterogeneity (e.g., study design) and statistical heterogeneity [23]. We used the I^2^ statistic to quantify the magnitude of statistical heterogeneity and high statistical heterogeneity was considered if I^2^ was > 75%.

Meta-analyses were performed when outcome-specific studies were sufficiently similar in terms of statistical heterogeneity (i.e. I^2^ ≤ 75%). We used random-effects models with the Mantel-Haenszel method to generate the pooled ORs and 95% CIs for outcomes. We performed prespecified subgroup analyses by study design (RCT vs. observational) for all outcomes. Finally, sensitivity analyses were conducted to assess the robustness of main findings by limiting to studies with the same gestational age at induction and studies with high quality. Additionally, leave-one-out meta-analysis was conducted for cohort studies by removing one study at a time to examine the influence of each individual study on the pooled estimate for primary outcomes. Potential publication bias was assessed graphically using funnel plots.

## Results

### Search results

**Fig. 1:**
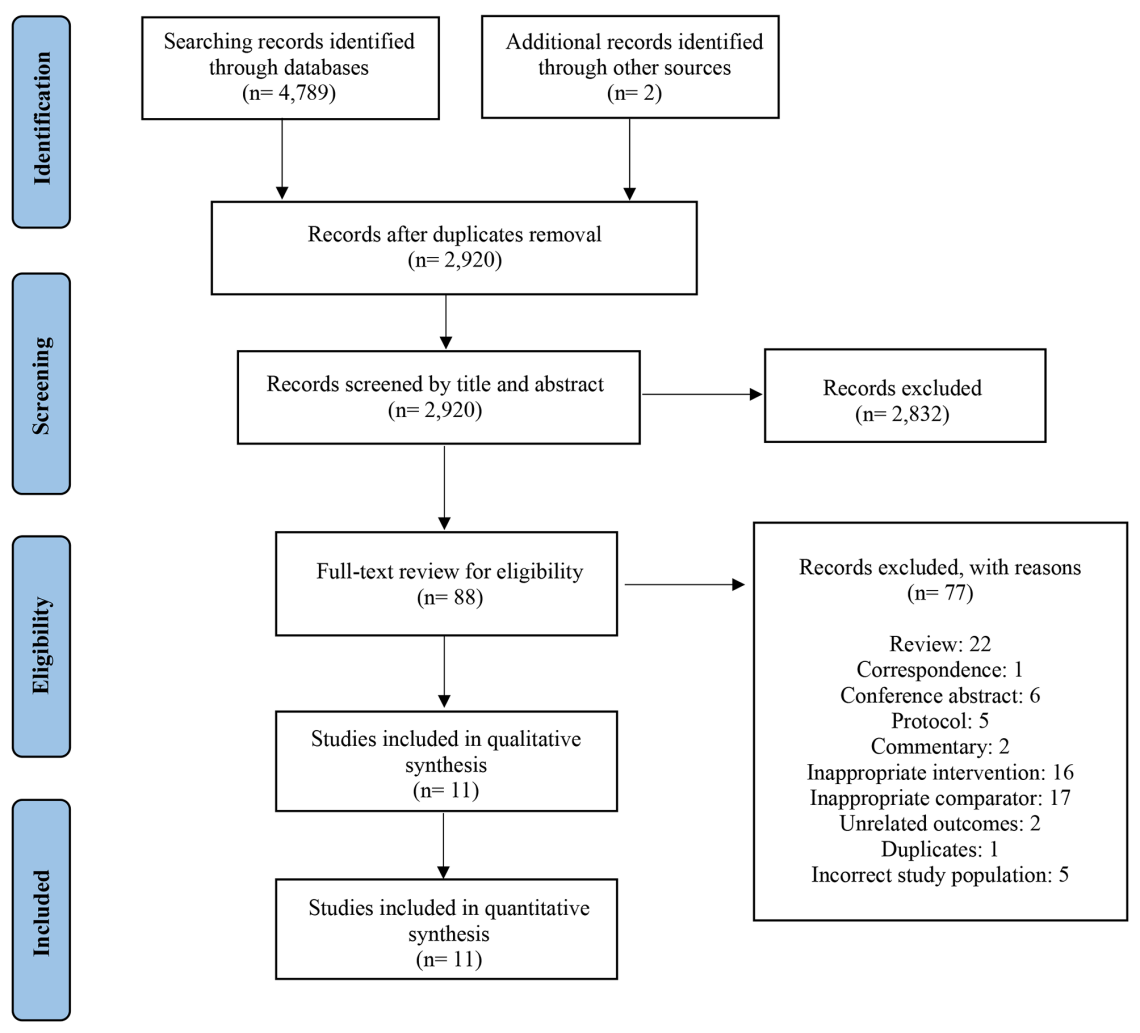
PRISMA flowchart diagram for study selection process

4,789 citations were identified by bibliographic database searches and an additional two citations were identified through manual searches of related reference lists and other sources (one masters dissertation [24] and one conference abstract [25]). Following de-duplication, 2,920 unique records remained. Through title and/or abstract review, 2,832 records were excluded for nonrelevance or not in English. The subsequent full-text screening against prespecified inclusion criteria further excluded 77 studies, leaving 11 for this systematic review and meta-analysis (Fig. 1) [12–14, 24, 26–32]. For the six conference abstracts included in the full-text review [25, 33–37], we contacted the original authors for additional details, however, all were ultimately excluded due to insufficient information for data extraction and methodological appraisal.

### Study characteristics

Descriptive information of the included studies is presented in Table 1 and Table S2. Eleven studies met the inclusion criteria [12–14, 24, 26–28, 30–32], resulting in 3,633 GDM women delivered following induction versus 9,984 GDM women with expectant management. The average age of participants ranged from 26 to 33 years. Studies were published between 1993 and 2019.

### General description of the RCTs

The three included RCTs were conducted in the US [26], India [24], and Italy [13], respectively. Two were performed in a single academic medical center [24, 26] and the other in multiple centers [13]. GDM diagnosis in two of the trials were based on the International Association of Diabetes and Pregnancy Study Groups (IADPSG) [13, 24], while the third didn’t report the diagnostic criteria (Table S2) [26]. Each trial used different study populations: insulin-requiring pre-gestational or GDM women [26], exclusively nutrition-controlled GDM [24], and women with both GDM subtypes (requiring nutrition therapy or pharmaceutical therapy) [13]. The trials were relatively small, with sample sizes ranging from 49 to 425 participants. Risk of bias was unclear for two trials [24, 26], and rated as high for the other (Fig. S1) [13].

### General characteristics of the observational studies

The effect of IOL in GDM term pregnancies was assessed in 7 cohort studies [12, 14, 27, 29–32] and 1 secondary analysis of an RCT [28]. Over half of these studies were conducted in the US [27–29, 31, 32], and the others in Canada [14], Italy [12] and Israel [30]. Of the observational studies, one included gestational and pre-gestational diabetic women [32], one only mild GDM [28], another only insulin-requiring GDM [29], and the remaining studies included both GDM subtypes [12, 14, 27, 30, 31]. All included studies but one [32] reported GDM diagnosis criteria based on a glucose challenge test (GCT) and/or oral glucose tolerance test (OGTT) (Table S2). More than half of the studies were small (< 400 subjects) or medium (400-1,000 subjects) in size. Risk of bias ranged from poor to high, with overall scores of 4 to 9 on a 9-point NOS scale (Table S3).

**Table 1:** Descriptive characteristics of the individual studies meeting inclusion criteria

First author & Publication year	Country & Study period	Study design	Sample size	Inclusion/exclusion criteria	Induction		Expectant management	Outcomes	Risk of bias ^c^
					Method	Timing ^b^			
Alberico et al., 2017 (13)	Italy 2010–2014	RCT	425	GDM women with a singleton, vertex presentation, gestational age at 38–39 weeks were eligible; Patients with previous CS, Bishop score > 7 contraindications to vaginal delivery, or an EFW of > 4000 g were excluded	Dinoprostone	38	IOL at 41^+ 0^ weeks	CS, instrumental vaginal delivery, severe perineal tears, ICU, macrosomia,5-min Apgar score, shoulder dystocia, NICU, perinatal mortality, neonatal acidemia	High
Kjos et al., 1993 (26)	USA 1987–1991	RCT	200	Women with uncomplicated, insulin-requiring diabetes at ≥ 38 weeks and with good blood glucose control were eligible; Patients with contraindications for a trial of vaginal delivery were excluded	Oxytocin/ prostaglandin	38	IOL if EFW ≥ 4200 g or ≥ 42 weeks	CS, LGA, macrosomia, shoulder dystocia, perinatal mortality	Unclear
Singh et al., 2013 (24)	India 2011–2012	RCT	52	Women with GDM well controlled by medical nutritional therapy at 39–40 weeks were eligible; Contradictions to vaginal delivery, suspected EFW > 3.5 kg or < 2.5 kg, multi-gestation, non-cephalic presentation were excluded	Misoprostol/ PGE1	39	IOL ≥ 40^6/7^ weeks	CS, instrumental vaginal delivery, severe perineal tear, ICU NICU, macrosomia, perinatal mortality, 5-min Apgar score, neonatal acidemia	Unclear
Alberico et al., 2010 (12)	Italy 1996–2006	Retrospective cohort	99	A1 and A2 GDM pregnancies with fetal growth acceleration at 38 weeks were eligible; an EFW ≥ 4 250 g or presence of another indication for elective CS were excluded	PGE2	38	CS if EFW > 4250 g	CS, macrosomia, NICU, shoulder dystocia, 5-min Apgar score, perinatal morality	Low
Melamed et al., 2016 (14)	Canada 2012–2014	Retrospective cohort	8392	All women with diagnosed GDM who had a singleton hospital birth at ≥ 38 weeks were eligible	NR ^a^	38–39	NR ^a^	CS, instrumental vaginal delivery, severe perineal tear, shoulder dystocia, NICU, perinatal mortality, LGA, macrosomia	Low
Vitner et al., 2019 (30)	Israel 2007–2014	Retrospective cohort	2472	GDM women with a singleton gestation who delivered at term were eligible; women with chronic medical conditions or contraindications for a trial of vaginal delivery were excluded	NR ^a^	37–40	IOL ≥ 40 weeks	CS, instrumental vaginal delivery, severe perineal tear, shoulder dystocia, perinatal mortality, LGA, macrosomia, 5-min Apgar score, acidemia, NICU	Low
Feghali et al., 2016 (27)	USA 2009–2012	Retrospective cohort	863	GDM women with singleton pregnancies, underwent IOL or spontaneous labour at ≥ 37 0/7 were eligible; those with a pre-labour indication for CS were excluded	NR ^a^	37–39	NR ^a^	CS	Low
Lurie et al., 1996 (29)	USA 1983–1994	Prospective Cohort	260	All insulin-requiring gestational diabetes women were eligible; Multi-gestation, breech presentation and complications of preeclampsia were excluded	Balloon catheter/PGE2/oxytocin	38	IOL if EFW > 4000 g	Shoulder dystocia, macrosomia, CS, instrumental vaginal delivery, perinatal mortality	High
Rayburn et al., 2005 (31)	USA 2002–2004	Retrospective cohort	280	GDM women requiring glyburide had a hospital singleton delivery at ≥ 37 weeks were eligible; Those with indications for pre-labour CS were excluded	Oxytocin	38	IOL if ≥ 42 weeks or fetal indications	Shoulder dystocia, CS, macrosomia, perinatal mortality, 5-min Apgar score	High
Sutton et al., 2014 (28)	USA 2002–2007	Secondary analysis of RCT	679	GDM women with cephalic presentation who delivered at term following induced or spontaneous labour were eligible; those had an scheduled CS or previous CS or major fetal anomaly were excluded	NR ^a^	37–40	NR ^a^	CS, perinatal mortality	Low
Conway et al., 1998 (32)	USA 1993–1995	Prospective cohort	2564	Gestational and pregestational diabetic patients with singleton, cephalic presentation deliveries were eligible; Deliveries before 35 weeks or with intrauterine fetal death were excluded	NR ^a^	LGA but EFW < 4250 g	NR ^a^	CS, shoulder dystocia, macrosomia, LGA	High

CS: caesarean section; ICU: intensive care unit; LGA: Large-for-gestational-age; NICU: neonatal intensive care unit;

^a^ Not reported; ^b^ timing of induction was according to gestational week or estimated fetal weight (EFW);^c^ Risk of bias was assessed by the Cochrane Risk of Bias Tool for RCTs and the Newcastle-Ottawa Scale for observational studies

### Effect of induction on CS

**Fig. 2:**
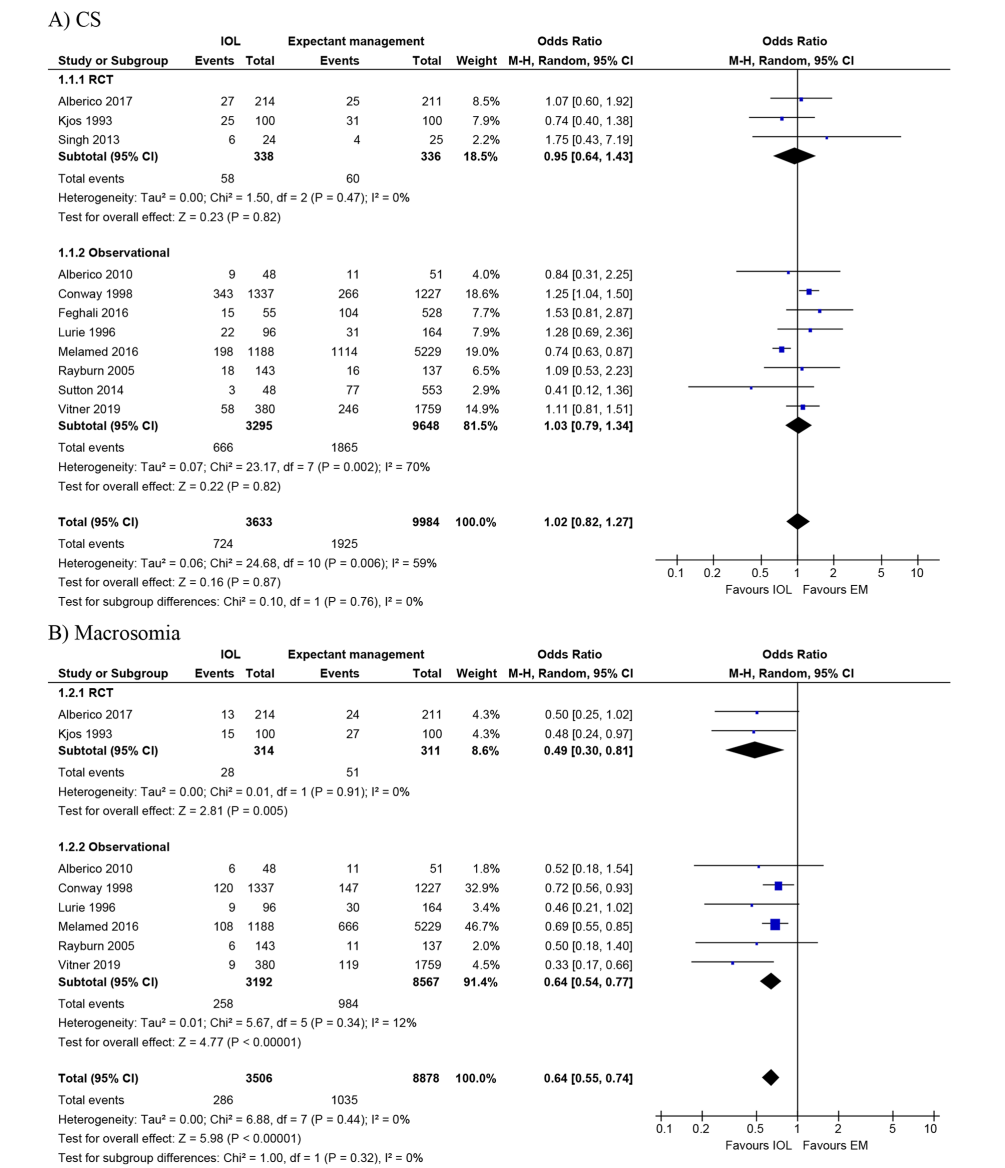
Forest plots for primary outcomes (**A**) CS (**B**) Macrosomia comparing IOL with expectant management in women with GDM at term gestation. Reference citations for studies can be found in Table 1. IOL, induction of labour; EM, expectant management

Eleven studies (3 RCTs [13, 24, 26] and 8 observational studies [12, 14, 27–32]) assessed CS following IOL versus expectant management among women with GDM (Table 2). Quantitatively, 10 of the 11 studies (3 RCTs [13, 24, 26] and 7 observational studies [12, 14, 27–31]) showed no evidence of harmful effect of IOL on CS: the crude effect estimates ranged from 0.41 to 1.75, among which one study reported a significantly decreased odds of CS among women undergoing induction compared to those having expectant management (0.74 [0.63–0.87]) [14]. One of the 11 studies found the odds of CS were increased 1.25-fold following induction compared to expectant management (1.25 [1.04–1.50]) [32]. Of the 3 RCTs that compared CS in women with GDM who underwent IOL (n = 338) versus expectant management (n = 336), the pooled odds of CS were not significantly different between groups (0.95 [0.64–1.43]; I^2^ = 0%). Similarly, meta-analysis of data from the 8 cohort studies [12, 14, 27–32] (n = 3,295 following IOL versus 9,648 expectant management) showed no clear difference in CS (1.03 [0.79–1.34]), but the studies were heterogeneous (I^2^ = 70%) (Fig. 2A). The GRADE quality of evidence rating was low for RCTs and very low for observational studies (Table S5).

### Effect of induction on macrosomia

Nine studies (3 RCTs [13, 24, 26] and 6 observational studies [12, 14, 29–32]) compared macrosomia in GDM women who underwent induction versus expectant management (n = 3,530 following IOL versus 8,903 expectant management) (Table 2). Four studies defined macrosomia as birth weight ≥ 4000 g [13, 26, 31, 32], and the remaining used birth weight > 4000 g [12, 14, 24, 29, 30]. Notably, one trial had no macrosomic neonates in both groups [24]. All studies consistently reported a lower rate of macrosomia associated with induction with the crude effect estimates ranging from 0.33 to 0.72, among which four studies reporting a significantly decreased odds of macrosomia [14, 26, 30, 32]. The pooled summary ORs showed IOL in women with GDM was associated with a reduction in macrosomia compared to expectant management (RCTs: 0.49 [0.30–0.81]; I^2^ = 0%; observational: 0.64 [0.54–0.77]; I^2^ = 0%) (Fig. 2B). The GRADE quality of evidence was low for RCTs and very low for observational studies (Table S5).

**Table 2:** Summary of pooled analyses of the effect of induction on adverse maternal and neonatal outcomes in women with GDM

Outcomes	Study design	*N* of studies		*N* of Women	Effect estimate (95% CI) ^i^	*I*^*2*^ (%)
Maternal outcomes						
CS	All		11	13,617	1.02 (0.82, 1.27)	59
	RCT		3	674	0.95 (0.64, 1.43)	0
	Observational		8	12,943	1.03 (0.79, 1.34)	70
Instrumental vaginal delivery	All		5	9290	0.99 (0.84, 1.17)	0
	RCT		2	474	0.84 (0.47, 1.50)	0
	Observational		3	8816	1.00 (0.85, 1.19)	0
Severe perineal lacerations ^a^	All ^b^		3	8829	**0.57 (0.38, 0.85)**	0
	RCT		1	273	0.13 (0.01, 2.52)	NA
	Observational		2	8556	**0.59 (0.39, 0.88)**	0
ICU	All		1	425	1.49 (0.25, 8.98)	NA
	RCT		1	425	1.49 (0.25, 8.98)	NA
Neonatal outcomes						
Macrosomia ^a^	All	8		12,384	**0.64 (0.55, 0.74)**	0
	RCT	2		625	**0.49 (0.30, 0.81)**	0
	Observational	6		11,759	**0.64 (0.54, 0.77)**	12
LGA	All	4		11,320	0.81 (0.63, 1.05)	61
	RCT	1		200	**0.37(0.17, 0.83)**	NA
	Observational	3		11,120	0.88 (0.72, 1.06)	42
Shoulder dystocia ^c^	All ^d^	7		12,232	0.80 (0.49, 1.31)	32
	RCT	2		625	0.75 (0.04, 15.49)	62
	Observational	5		11,607	0.79 (0.49, 1.30)	36
NICU ^a^	All	4		9080	1.40 (0.90, 2.19)	59
	RCT	1		425	0.99 (0.14, 7.06)	NA
	Observational	3		8655	1.41 (0.85, 2.32)	72
5-min Apgar score < 7 ^a^	All ^e^	4		2943	0.61 (0.17, 2.22)	7
	RCT	1		425	4.98 (0.24, 104.28)	NA
	Observational	3		2518	0.40 (0.10, 1.55)	0
Neonatal acidemia ^a^	All ^f^	2		2564	1.84 (0.38, 8.92)	14
	RCT	1		425	7.00 (0.36, 136.35)	NA
	Observational	1		2139	1.16 (0.24, 5.48)	NA
Perinatal mortality ^g^	All ^h^	5		9195	0.64 (0.16, 2.58)	0
	Observational	5		9195	0.64 (0.16, 2.58)	0

CS: caesarean section; ICU: intensive care unit; LGA: Large-for-gestational-age; NICU: neonatal intensive care unit;

^a^ One RCT (Singh et al. 2013) had no events recorded in both induction group and expectant management and were excluded from the final pooled analyses for severe perineal lacerations, macrosomia, NICU, 5-min Apgar score < 7 and neonatal academia; ^b^ One RCT (Alberico et al. 2017) and one observational study (Vitner et al. 2019) had no events recorded in one group and applied a fix correction approach; ^c^ One observational study (Alberico et al. 2010) had no events recorded in both groups and were excluded from the final pooled analyses for shoulder dystocia; ^d^ One RCT (Kjos et al. 1993) had no events recorded in one group and applied a fix correction approach; ^e^ One RCT (Alberico et al. 2017) and two observational studies (Alberico et l 2010; Vitner et al. 2019) had no events recorded in one group and applied a fix correction approach; ^f^ One RCT (Alberico et al. 2017) had no events recorded in one group and applied a fix correction approach; ^g^ Three RCTs (Kjos et al. 1993; Singh et al. 2013; Alberico et al. 2017) and one observational study (Sutton et al. 2014) had no recorded events in both groups and were excluded from the final pooled analyses for perinatal mortality; ^h^ Five observational studies (Alberico et al. 2010; Lurie et al. 1996; Melamed et al. 2016; Rayburn et al. 2005; Vitner et al. 2019) had no events recorded in one group and applied a fix correction approach; ^i^ Pooled odds Ratio (OR) was generated by random-effects models with the Mantel-Haenszel method

### Effect of induction on secondary outcomes

The combined results for severe perineal lacerations from two observational studies significantly favored IOL (0.59 [0.39–0.88]; I^2^ = 0%) while no significant association was demonstrated in the single trial (0.13 [0.01–2.52]). For LGA, the trial showed a protective effect of induction (0.37 [0.17–0.83]) but no significant difference was observed in the pooled results from 3 observational studies (0.88 [0.72–1.06]; I^2^ = 42%). Additionally, compared with expectant management, induction in GDM women was not significantly associated with instrument vaginal delivery (RCTs: 0.84 [0.47–1.50]; I^2^ = 0%; observational: 1.00 [0.85–1.19]; I^2^ = 0%), ICU admission (RCT: 1.49 [0.25–8.98]), shoulder dystocia (RCTs: 0.75 [0.04–15.49]; I^2^ = 62%; observational: 0.79 [0.49–1.30]; I^2^ = 36%), NICU (RCT: 0.99 [0.14–7.06]; observational: 1.41 [0.85–2.32]; I^2^ = 72%), 5-min Apgar score < 7 (RCT: 4.98 [0.24-104.28]; observational: 0.40 [0.10–1.55]; I^2^ = 0%), neonatal acidemia (RCT: 7.00 [0.36-136.35]; observational: 1.16 [0.24–5.48]) and perinatal mortality (0.64 [0.16–2.58]; I^2^ = 0%) (Table 2; Fig. S2-S10). The GRADE quality of evidence rating for the secondary outcomes ranged from very low to low (Table S5).

### Publication bias and sensitivity analysis

We assessed publication bias using funnel plots and no obvious asymmetry was evident for the primary outcomes (Fig. S11). The results of the sensitivity analyses by limiting studies to high quality or induction at same gestational age for the primary outcomes were similar to the main results (Fig. S12-S13). The leave-one-out sensitivity analyses showed similar pooled effect estimates and found Melamed’s study had a key contribution to the between-study heterogeneity and the overall effect of induction on primary outcomes (Table S4).

## Discussion

### Main findings

Our study found that comparative studies of adverse maternal and perinatal outcomes following induction at term in women with GDM are limited in number and have yielded inconsistent results. This meta-analysis quantitatively assessed the effect of induction and shows that among women with GDM, induction at term is associated with lower odds of macrosomia and severe perineal lacerations than expectant management, but no evidence of significant difference in CS between groups was found. No significant between-group differences were observed in other maternal or neonatal morbidities, or perinatal mortality. The overall quality of evidence across studies was low to very low due to limited and inconsistent findings from a few studies with mixed study design, precluding our ability to draw firm conclusions.

### Strengths and limitations

This systematic review and meta-analysis used a comprehensive literature search strategy. We included all related comparative studies published on a topic of highly relevant to obstetric clinical decision-making. The risk of bias assessment and data extraction were done independently and in duplicates to minimize assessor bias. Overall, the 11 included studies collectively enrolled over 13,000 women with GDM to answer the research question. To our knowledge, no prior systematic review and meta-analysis on this topic is as extensive and comprehensive as ours, which has quantitatively assessed the effect of induction on various outcomes in GDM women using data from different populations. The review included both randomized and observational study designs to maximize analysis of all available evidence.

Nevertheless, there are several limitations. The most important limitation was the small number of well-designed, adequately powered studies reporting maternal and neonatal outcomes of interest: there were only 3 RCTs, 1 of which had low quality, and the other 2 had unclear risk of bias. As there were no more than 5 studies for most of outcomes, quantitative synthesis may have introduced between-study heterogeneity and affected the validity of pooled results. Among the 11 studies that met inclusion criteria, most were small studies and had imprecise estimates. With the leave-one-out sensitivity analysis, we identified one study with a relatively larger impact on the pooled effect estimates for the primary outcomes [14]. Another particular concern was that the included studies were from a wide range of time periods, over which the diagnostic criteria for GDM, induction methods, and obstetrical practice patterns had changed. This may, at least in part, explain the heterogeneity for several outcomes. Additionally, most of the included women were GDM patients with different severity, and the lack of reporting of stratified data of GDM subtypes in original studies prevented us from conducting subgroup analyses by GDM severity. Observational studies, which accounted a substantial proportion of study participants in this study, may be prone to a certain degree of bias that could affect the validity. These include the use of non-concurrent or incomparable control groups in some included studies [29, 31, 32] which may introduce selection bias and exaggerate the observed association away from the null. Expectant management was defined consistently across studies as women who delivered following spontaneous or IOL at later gestational ages, with the exception of one study including spontaneous vaginal delivery at the same week as the induction group [28]. Therefore, the pooled effect estimate for CS might be biased slightly away from the null. Because we do not have access to raw data of the original studies, and given the differences in contents and format of confounding variables in original studies, the lack of adjustment for important confounders including glycemic control in our meta-analysis also suggests a possibility of residual confounding, which might limit the interpretation of the study findings. The exclusion of non-English language studies in this study might also create language bias. Lastly, publication bias may still exist in the case of small negative unpublished studies. Although it was not detected in this study, the insufficient number of included studies limits the power to detect publication bias [38].

### Interpretation

In view of the recommendations from many clinical guidelines worldwide that women with GDM should be offered induction at term, the trade-off of benefits and risks of this intervention is of high significance for decision-making by healthcare providers and patients in practice. In this systematic review and meta-analysis, we found induction at term gestation for GDM women was associated with lower odds of macrosomia and severe perineal lacerations compared to expectant management, but no significant differences in CS, instrumental vaginal delivery, LGA or other severe maternal and neonatal outcomes.

The finding of non-significant difference in CS from this updated systematic review and meta-analysis is similar to the results from previous reviews [15–17]. However, the results from the Cochrane review of 1 trial were limited to insulin-treated diabetic pregnant women [17]. An earlier systematic review of five studies evaluating the effect of induction in GDM pregnancies was limited by high heterogeneity and methodological concerns, precluding a quantitative synthesis [15]. The non-significant difference in CS between groups in our study is likely due to a considerable amount of GDM women expectantly managed ultimately requiring induction or CS at later gestational ages for other indications (e.g. post-term). Additionally, with the increasing fetal weight, aging placenta and decreasing amniotic fluid associated with advancing pregnancy, women having expectant management might therefore be predisposed to have intrapartum CS due to non-reassuring fetal heart rates [39, 40]. Our finding of a significantly lower odds of macrosomia associated with induction was conflicting with the results from a recent Cochrane review of one trial by Biesty et al., which was an open-label trial including 425 subjects [16]. With exclusion of GDM subjects with suspected macrosomia and severe recruiting challenges [13], it was unable to achieve the target sample size, and therefore underpowered to identify between-group differences [13, 16]. The lower likelihood of macrosomia in GDM pregnancies with induction in our study might be explained based on the modified Pedersen hypothesis of maternal hyperglycemia leading to fetal hyperinsulinemia and increased glucose utilization [41]. Consequently, it results in fetal excess protein and fat storage, leading to macrosomia. Moreover, as fetal growth is positively associated with advancing gestational age, expectant management extends the gestation of GDM pregnancy and boosts intrauterine fetal growth, which may further increase the risk of macrosomia [42]. This finding was also supported by recent evidence of elective induction associated with a lower neonatal birth weight among low-risk pregnancies [40].

Nevertheless, we couldn’t draw a definite conclusion to guide decisions given the small number and overall low quality of the eligible studies. Results of secondary outcomes, especially for those related to severe neonatal morbidities and perinatal mortality, were underpowered given the rare nature of these outcomes.

Labour and delivery management for pregnancies with GDM, especially with macrosomia, remains an obstetric challenge. Emerging evidence has shed light on the criticality of delivery timing and mode in such pregnancies to maximize health outcomes considering the common tools used for pregnancies monitoring and antenatal fetal surveillance at term, such as ultrasonography, fetal cardiotocography (CTG) and Doppler studies of umbilical artery (UA), exhibit limited utility in the context of GDM and macrosomia [43, 44]. This is primarily due to the unique fetal growth patterns and increased risk of complications associated with GDM and macrosomia [42, 45, 46]. Consequently, there is a growing recognition of the need to necessitate tailored and individualized approaches to deliver these pregnancies which take into account maternal glycemic control status, fetal well-being, and the potential risks linked with prolonged intrauterine exposure to elevated glycemic levels. Comprehensive and well-designed comparative studies with large sample sizes, assessing a wide range of adverse outcomes, are required to generate robust evidence and inform clinical guidance to improve labour and delivery management in GDM pregnancies.

## Conclusions

In this systematic review and meta-analysis, we found induction at term gestation in GDM women may reduce the risk of macrosomia and severe perineal lacerations compared to expectant management, but the effect on CS or severe maternal and neonatal outcomes was inconclusive. Definitive evidence from well-designed studies with large sample sizes are warranted to better inform implications for labour and delivery management in women with GDM.

## Electronic supplementary material

Below is the link to the electronic supplementary material.

Supplementary Material 1: Appendix S1

Supplementary Material 2: Table S2

Supplementary Material 3: Figure S1

Supplementary Material 4: Table S3

Supplementary Material 5: Table S5

Supplementary Material 6: Figure S2

Supplementary Material 7: Figure S3

Supplementary Material 8: Figure S4

Supplementary Material 9: Figure S5

Supplementary Material 10: Figure S6

Supplementary Material 11: Figure S7

Supplementary Material 12: Figure S8

Supplementary Material 13: Figure S9

Supplementary Material 14: Figure S10

Supplementary Material 15: Figure S11

Supplementary Material 16: Figures S12 and S13

Supplementary Material 17: Table S4

Supplementary Material 18: Table S1

## Data Availability

All data generated or analysed during this study are included in this published article and its supplementary information files.

## List of Abbreviations

GDM: Gestational diabetes mellitus
IOL: Induction of labour
SOGC: Society of Obstetricians and Gynaecologists of Canada
DC: Diabetes Canada
NICE: National Institute for Health and Care Excellence
ACOG: American College of Obstetricians and Gynecologists
PRISMA: Preferred Reporting Items for Systematic Reviews and Meta-analyses
MeSH: Medical Subject Headings
ICTRP: International Clinical Trials Registry Platform
PICOS: Population-Intervention-Comparators-Outcomes-Study design
RCT: Randomized controlled trial
CS: Caesarean section
LGA: Large-for-gestational-age
ICU: Intensive care unit
NICU: Neonatal intensive care unit
NOS: Newcastle-Ottawa Scale
OR: Odds ratio
CI: Confidence intervals
GCT: Glucose challenge test
OGTT: Oral glucose tolerance test
GRADE: Grading of Recommendations Assessment, Development, and Evaluation
CTG: Cardiotocography
UA: Umbilical artery
