# Supplementary material for: Comparison of adverse maternal and perinatal outcomes between induction and expectant management among women with gestational diabetes mellitus at term pregnancy: a systematic review and meta-analysis
Source: BMC Pregnancy Childbirth. 2023 Jul 12;23:509. doi: 10.1186/s12884-023-05779-z (PMC10339546; doi:10.1186/s12884-023-05779-z)
Supplement: Supplementary file 1 — Supplementary Material 1: Appendix S1 [file 12884_2023_5779_MOESM1_ESM.docx]

***Additional information***

**Polyhydroxybutyrate Production by Recombinant *Escherichia coli* Based on Genes Related to Synthesis Pathway of PHB from *Massilia* sp. UMI-21**

**Nan Jiang, Ming Wang, Linxin Song, Dengbin Yu, Shuangzi Zhou, Yu Li, Haiyan Li and Xuerong Han^*^**

*** Correspondence:** Xuerong Han: hanxuerong@jlau.edu.cn

**Table S1** PHB-producing strains in the genus *Massilia*.

| **Strain** | **Carbon source** | **Medium** | **Fermentation time (h)** | **PHB content (wt%)** | **Reference** |
| --- | --- | --- | --- | --- | --- |
| *M. albidiflava* | starch | ISP2 | 48 | ~ 26 | [1] |
| *M. dura* | starch | ISP2 | 48 | ~ 14 | [1] |
| *M. lutea* | starch | ISP2 | 48 | ~ 33 | [1] |
| *M. aerilata* | starch | ISP2 | 48 | ~ 33 | [1] |
| *M. plicata* | starch | ISP2 | 48 | < 1 | [1] |
| *M. brevitalea* | starch | ISP2 | 48 | ~ 16 | [1] |
| *M. aurea* | starch | ISP2 | 48 | ~ 32 | [1] |
| *Massilia* sp. 2C4 | glucose | OM | 48 | 5.4 ± 2.4 | [2] |
| *Massilia* sp. 4A1 | glucose | OM | 48 | 10.2 ± 2.3 | [2] |
| *Massilia* sp. 4D3c | glucose | OM | 48 | 2.9 ± 1.8 | [2] |
| *Massilia* sp. 4D6 | glucose | OM | 48 | 45.7 ± 2.4 | [2] |
| *Massilia* sp. 5F6 | glucose | OM | 48 | < 1 | [2] |
| *Massilia* sp. 5F8 | glucose | OM | 48 | 6.9 ± 1.6 | [2] |
| *M. umbonata* LP01^T^ | soluble starch | ISP2 | 72 | 22.6 | [3] |
| *Massilia* sp. UMI-21 | soluble starch | nitrogen-limiting MS | 72 | 27.2 | [4] |

**Table S2.** Primers used in recombinant plasmid construction.

| **Target gene** | **Primer name** | **Primer sequence (5'–3')** | Amplicon size (bp) |
| --- | --- | --- | --- |
| *phaA2B1* | A2B1F-PstI | TCGAGCTCGGCGCGCctgcagATGGAAGATGTCGTCATCGTG | 2006 |
|  | A2B1R-SalI | TGCGGCCGCAAGCTTgtcgacTTACTGCATGTGCTGCCCAC |  |
| *phaC1* | C1F-NdeI | TAAGAAGGAGATATAcatatgATGCCTGACCCCCAAGCTT | 1734 |
|  | C1R-EcoRV | ATCGCGTGGCCGGCCgatatcTTATTCCGCGCGCGCCTT |  |
| *vgb* | vgbF-PacI | GTCTACTAGCGCAGCttaattaaATGCTGGATCAGCAGACCATTAA | 441 |
|  | vgbR-AvrII | CAGCGGTGGCAGCAGcctaggTTATTCCACTGCCTGTGCATACA |  |

Primer sequence consists of: 5’-HOMOLOGOUS SEQUENCE OF VECTOR pETDuet1-restriction enzyme cutting site-TARGET GENE SPECIFIC PRIMER SEQUENCE-3’.


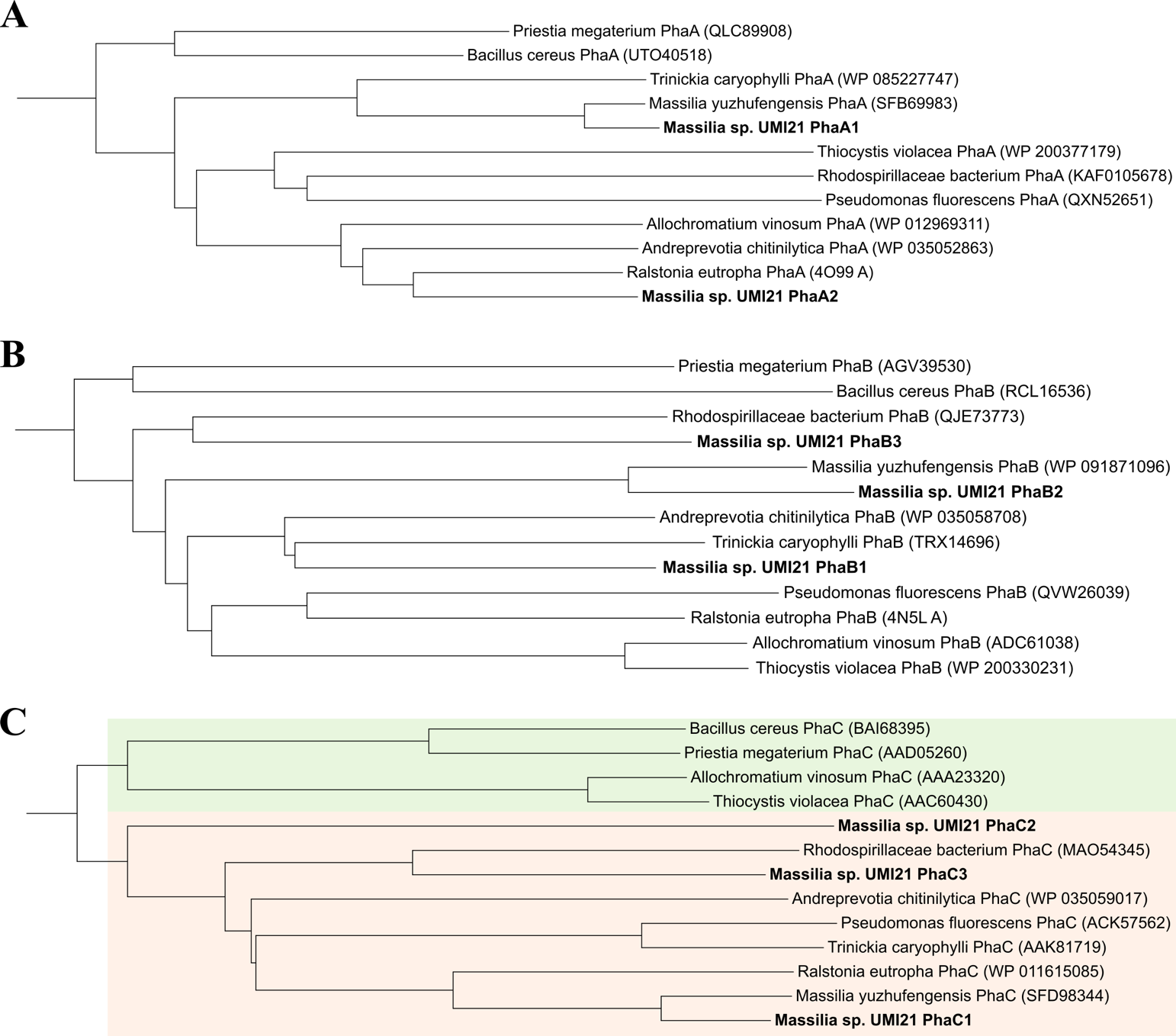


**Fig. S1** Phylogenetic tree of PHA-related proteins from *Massilia* sp. UMI-21 and related taxa. **(A)** PhaA. **(B)** PhaB. **(C)** PhaC. The GenBank accession numbers were shown in the parentheses. Green denotes Class III and IV PhaC; orange denotes Class I and II PhaC.


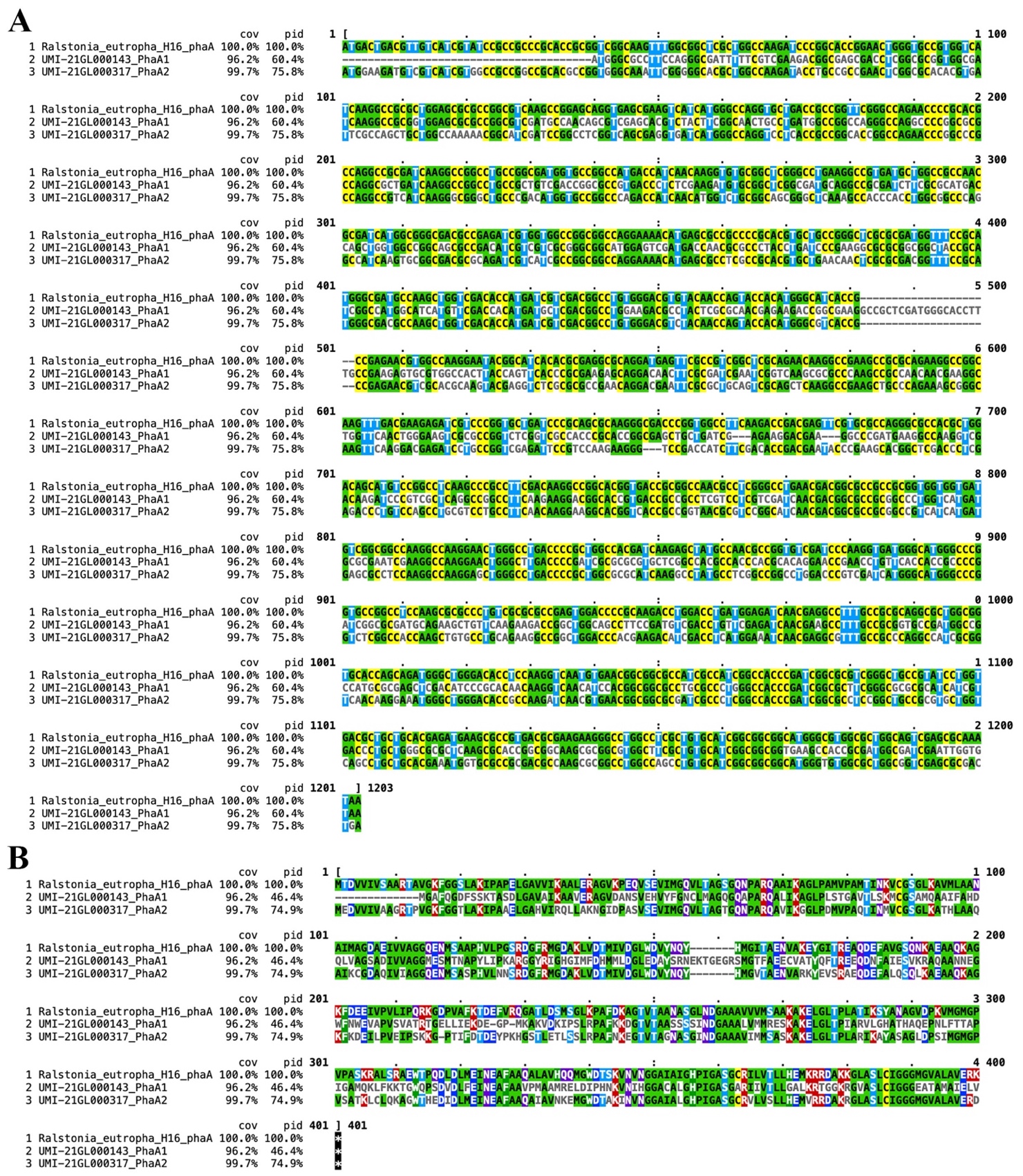


**Fig. S2** Results of *phaA* gene and amino acid (AA) sequence alignment between *Ralstonia eutropha* H16 and *Massilia* sp. UMI-21. **(A)** Alignment of DNA sequences. **(B)** Alignment of AA sequences.


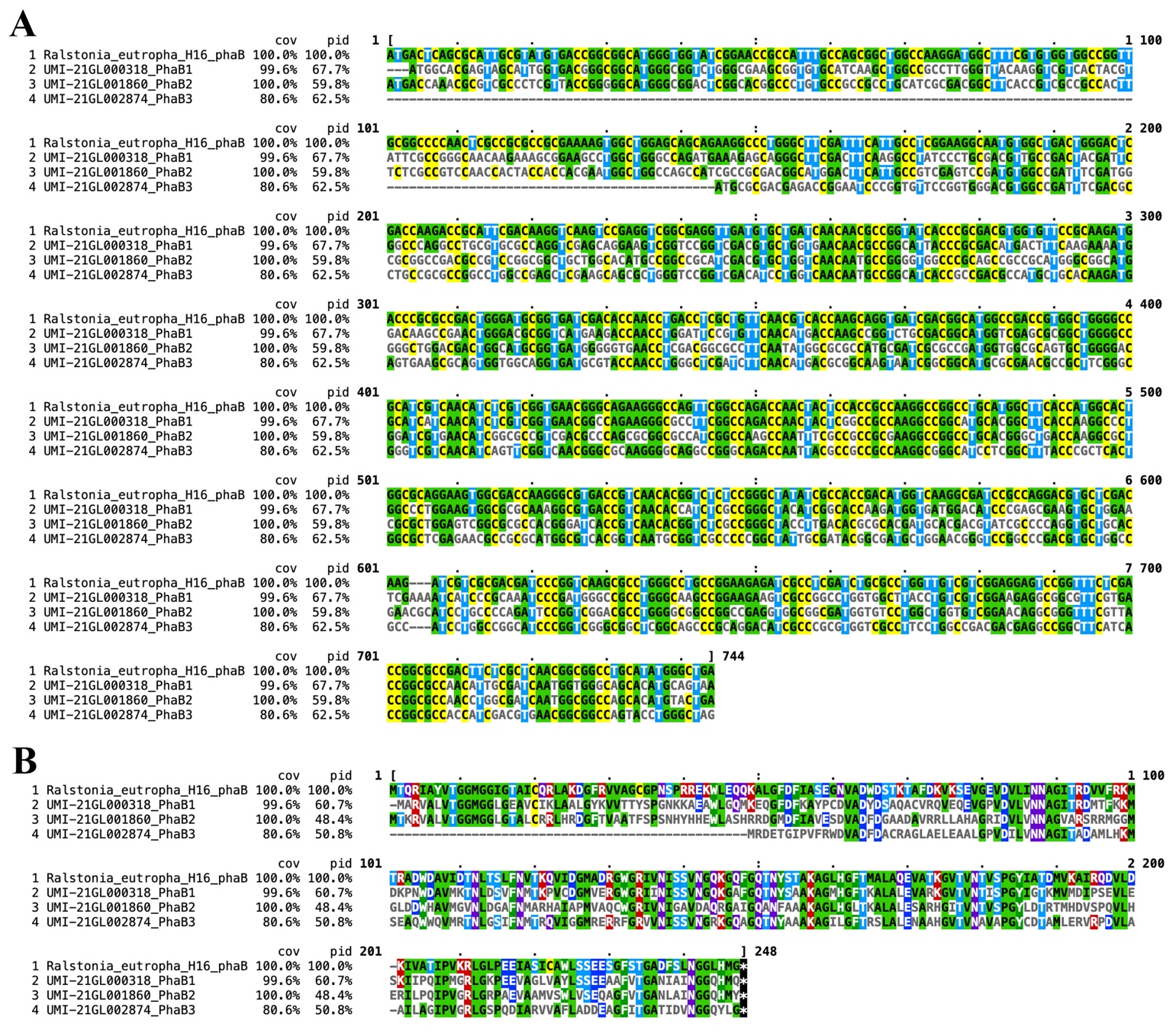


**Fig. S3** Results of *phaB* gene and amino acid (AA) sequence alignment between *Ralstonia eutropha* H16 and *Massilia* sp. UMI-21. **(A)** Alignment of DNA sequences. **(B)** Alignment of AA sequences.


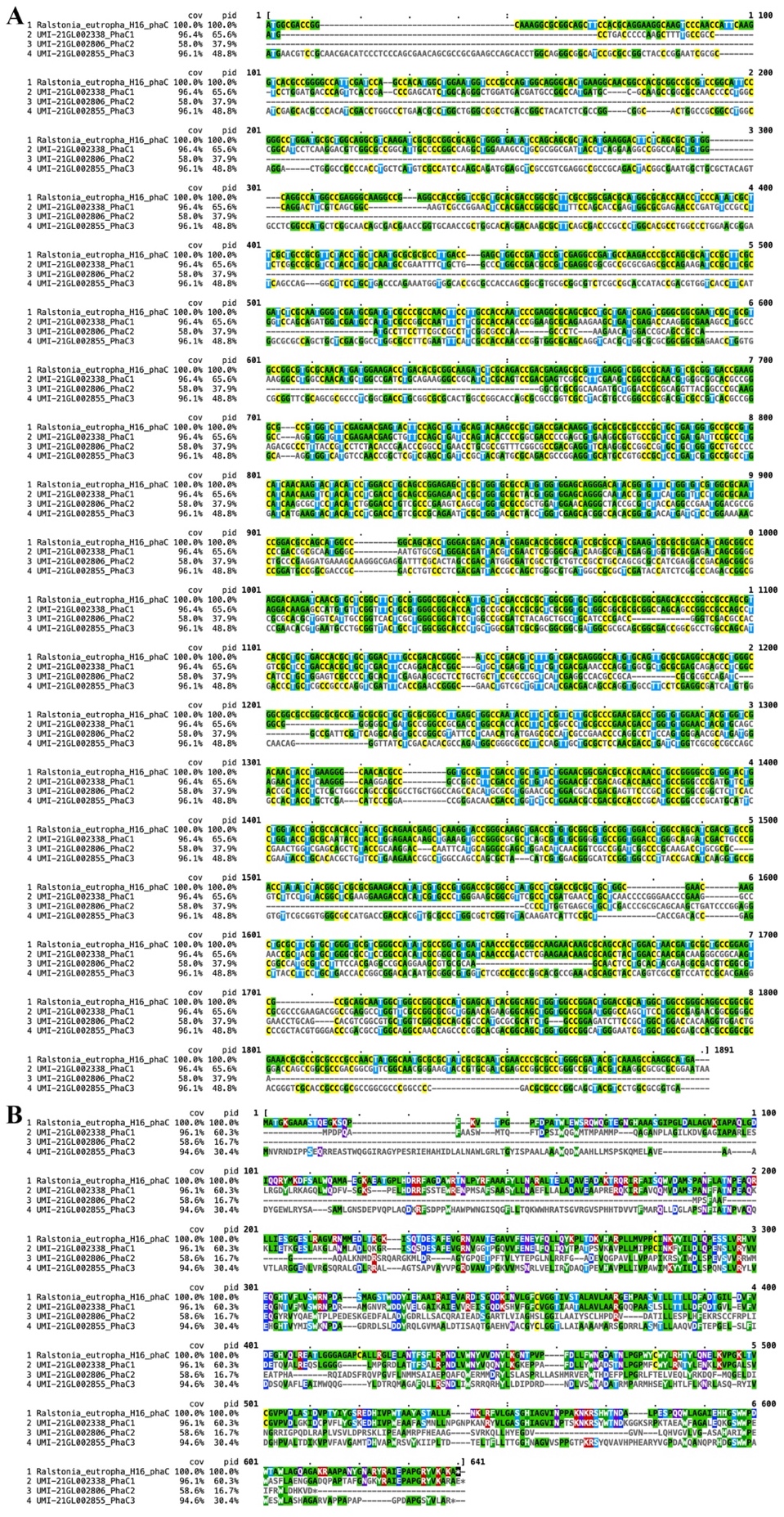


**Fig. S4** Results of *phaC* gene and amino acid (AA) sequence alignment between *Ralstonia eutropha* H16 and *Massilia* sp. UMI-21. **(A)** Alignment of DNA sequences. **(B)** Alignment of AA sequences.


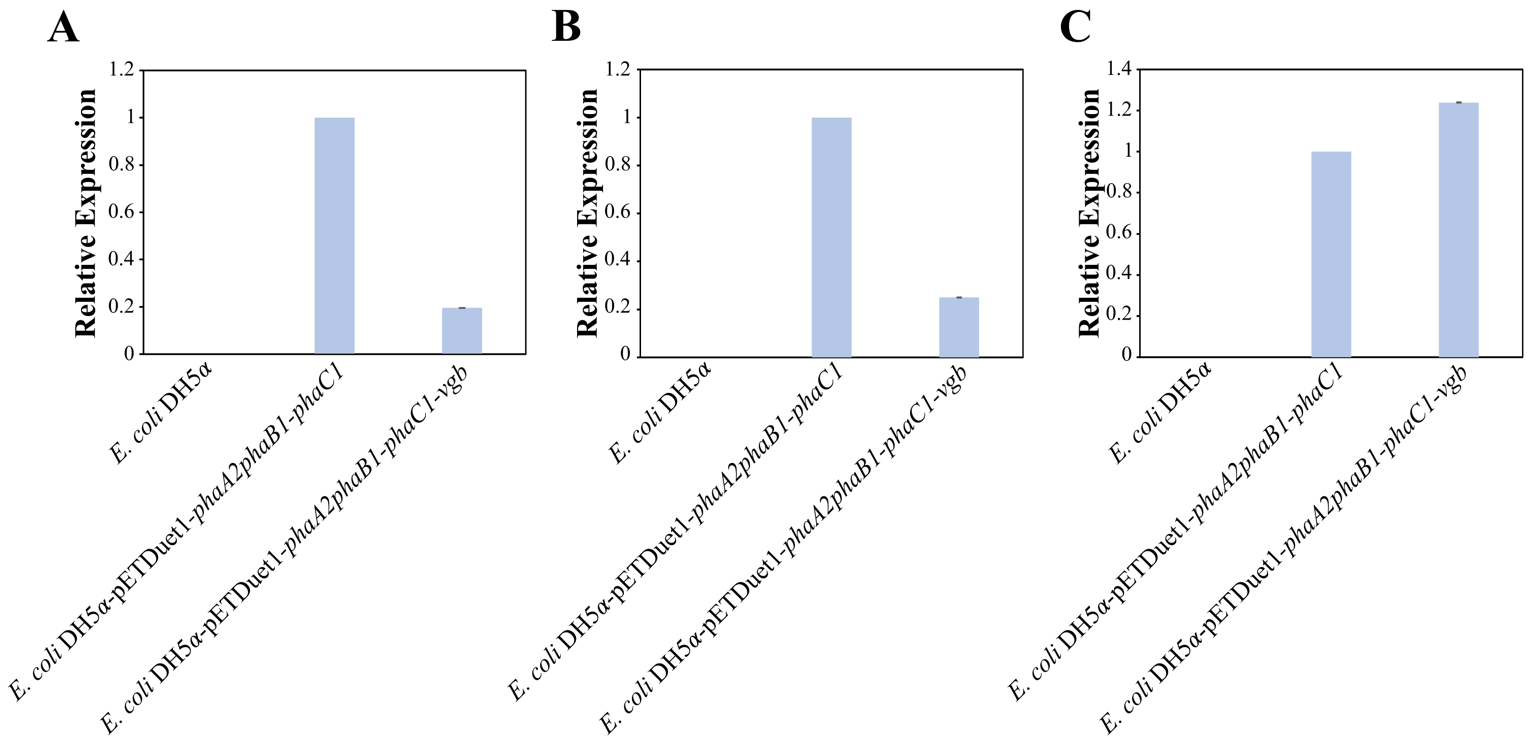


**Fig. S5** Relative expression of *phaA2* **(A)**, *phaB1* **(****B)** and *phaC1* **(C)** genes in wild *E. coli* DH5α strain and the recombinant *E. coli* DH5α-pETDuet1-*phaA2phaB1-phaC1* and DH5α-pETDuet1-*phaA2phaB1-phaC1-vgb* strains by qRT-PCR. 16S rRNA serves as the internal control.

**References**

1. Cerrone F, Sánchez-Peinado MdM, Rodríguez-Díaz M, González-López J, Pozo C. PHAs production by strains belonging to *Massilia* genus from starch. Starke 2011;63:236-240.

2. Bassas-Galia M, Nogales B, Arias S, Rohde M, Timmis KN, Molinari G. Plant original *Massilia* isolates producing polyhydroxybutyrate, including one exhibiting high yields from glycerol. J Appl Microbiol 2012;112:443-454.

3. Rodríguez-Díaz M, Cerrone F, Sánchez-Peinado M, SantaCruz-Calvo L, Pozo C, López JG. *Massilia umbonata* sp. nov., able to accumulate poly-β-hydroxybutyrate, isolated from a sewage sludge compost-soil microcosm. Int J Syst Evol Microbiol 2014;64:131-137.

4. Han X, Satoh Y, Kuriki Y, Seino T, Fujita S, Suda T, Kobayashi T, Tajima K. Polyhydroxyalkanoate production by a novel bacterium *Massilia* sp. UMI-21 isolated from seaweed, and molecular cloning of its polyhydroxyalkanoate synthase gene. J Biosci Bioeng 2014;118:514-519.
