# Supplementary material for: Comparison of adverse maternal and perinatal outcomes between induction and expectant management among women with gestational diabetes mellitus at term pregnancy: a systematic review and meta-analysis
Source: BMC Pregnancy Childbirth. 2023 Jul 12;23:509. doi: 10.1186/s12884-023-05779-z (PMC10339546; doi:10.1186/s12884-023-05779-z)
Supplement: Supplementary file 2 — Supplementary Material 2: Table S2 [file 12884_2023_5779_MOESM2_ESM.docx]

**Table S2.** Further characteristics of the included studies

| **Study** | **Study Population** | | | **GDM subtypes** | **GDM Diagnosis criteria** | **Timing of diagnosis (weeks)** | **Measurement of gestational age** |
| --- | --- | --- | --- | --- | --- | --- | --- |
|  | **Maternal age (y) ^a^** | **Nulliparity ^b^** | **Obesity ^c^** |  |  |  |  |
| Alberico et al.  2017 | 32.3 ± 6.1  32.4 ± 5.6 | 127 (59.4)  104 (49.3) | NR ^d^ | A1GDM/A2GDM | IADPSG | NR ^d^ | LMP and Ultrasonography |
| Kjos et al.  1993 | 32.1 ± 0.61  31.9 ± 0.56 | NR ^d^ | NR ^d^ | A2GDM/Pre-existing DM | NR ^d^ | NR ^d^ | LMP and Ultrasonography |
| Singh et al.  2013 | 26.0 ± 4.9  26.1 ± 4.22 | 14 (58.0)  12 (48.0) | NR ^d^ | A1GDM | IADPSG | NR ^d^ | LMP and Ultrasonography |
| Alberico et al.  2010 | 33.3 ± 4.9  32.7 ± 5.1 | 39 (63.0)  30 (59.0) | 18 (37)  11 (21) | A1GDM/A2GDM | 2000-2010 ADA diagnostic criteria | 24-28 | NR ^d^ |
| Melamed et al.  2016 | 32.5 ± 5.1  32.5 ± 5.0 | 483 (40.7)  2632 (50.3) | 529 (44.5)  1650 (31.5) | A1GDM/A2GDM | 2008 and 2013 CDA guideline | NR ^d^ | NR ^d^ |
| Vitner et al.  2019 | 33.0 ± 4.8  32.7 ± 4.8 | 150 (39.5)  670 (38.1) | NR ^d^ | A1GDM/A2GDM | 2001 ACOG diagnostic criteria | 24-28 | LMP and Ultrasonography |
| Feghali et al.  2016 | 31.0 ± 5.2 | 90 (48.9) | NR ^d^ | A1GDM /A2GDM | CC criteria | NR ^d^ | NR ^d^ |
| Lurie et al.  1996 | 32.5 ± 6.1  31.1 ± 5.0 | NR ^d^ | NR ^d^ | A2GDM | O’Sullivan and Mahlan | NR ^d^ | LMP and Ultrasonography |
| Rayburn et al.  2005 | 30.0 ± 6.5  30.0 ± 6.8 | 26 (18.0)  42 (31.0) | NR ^d^ | A1GDM/A2GDM | ACOG diagnostic criteria | NR ^d^ | Ultrasonography |
| Sutton et al.  2014 | 28.9 ± 5.5 | 38 (26.2) | NR ^d^ | Mild GDM | CC Criteria | 24-30 | NR ^d^ |
| Conway et al.  1998 | 28.5 ± 6.6  28.8 ± 6.8 | NR ^d^ | NR ^d^ | GDM/Pre-existing DM | NR ^d^ | NR ^d^ | NR ^d^ |

^a^ presented as mean ± SD for intervention and control groups; ^b^ presented as number (%) for intervention and control groups; ^c^ refers to maternal BMI >30 mg/m^2^, presented as number (%) for intervention and control groups; ^d^ NR: Not reported;

LMP: Last menstrual period; ADA: American Diabetes Association; CC: Carpenter and Coustan criteria; IADPSG: The International Association of Diabetes and Pregnancy Study Groups (IADPSG) criteria; CDA: Canadian Diabetes Association; ACOG: American Committee of Obstetrics and Gynaecology;
