# Supplementary material for: Comparison of adverse maternal and perinatal outcomes between induction and expectant management among women with gestational diabetes mellitus at term pregnancy: a systematic review and meta-analysis
Source: BMC Pregnancy Childbirth. 2023 Jul 12;23:509. doi: 10.1186/s12884-023-05779-z (PMC10339546; doi:10.1186/s12884-023-05779-z)
Supplement: Supplementary file 3 — Supplementary Material 3: Figure S1 [file 12884_2023_5779_MOESM3_ESM.docx]

(A)

(B)

**Fig. S1** Assessment of risk of bias for RCTs. (A) Summary of risk of bias (+: low risk of bias; -: high risk of bias; ?: unclear risk of bias); (B) Risk of bias graph with each risk of bias item presented as percentages across all included trials
