# Supplementary material for: Comparison of adverse maternal and perinatal outcomes between induction and expectant management among women with gestational diabetes mellitus at term pregnancy: a systematic review and meta-analysis
Source: BMC Pregnancy Childbirth. 2023 Jul 12;23:509. doi: 10.1186/s12884-023-05779-z (PMC10339546; doi:10.1186/s12884-023-05779-z)
Supplement: Supplementary file 4 — Supplementary Material 4: Table S3 [file 12884_2023_5779_MOESM4_ESM.docx]

**Table S3**. Quality assessment for the observational studies

| Study | Selection | | | | Comparability | | Outcome | | | NOS Score |
| --- | --- | --- | --- | --- | --- | --- | --- | --- | --- | --- |
|  | 1 | 2 | 3 | 4 | 5_a_ | 5_b_ | 6 | 7 | 8 |  |
| Alberico et al, 2010 | * | * | * | * | * | * | * | * | * | 9 |
| Feghali et al, 2016 | * | * | * | * | * | * | * | * | * | 9 |
| Lurie et al, 1996 | * | - | - | * | - | - | * | * | * | 5 |
| Melamed et al, 2016 | * | * | * | - | * | * | * | * | * | 8 |
| Rayburn et al, 2005 | * | - | * | * | - | - | * | * | * | 6 |
| Sutton et al, 2014 | * | * | - | * | * | * | - | * | * | 7 |
| Vitner et al, 2019 | * | * | * | * | * | * | * | * | * | 9 |
| Conway et al, 1998 | * | - | - | * | - | - | - | * | * | 4 |

NOS: Newcastle-Ottawa Scale;

1. Representativeness of the exposed cohort; 2. Selection of the non-exposed cohort; 3. Ascertainment of exposure; 4. Demonstration that outcome of interest was not present at start of study; 5_a_ Comparability of cohort: control selected (the most important factor); 5_b_ study controls for additional factor; 6. Assessment of outcome; 7. long enough follow- up for outcomes; 8. Adequacy of follow-up
