# Supplementary material for: Comparison of adverse maternal and perinatal outcomes between induction and expectant management among women with gestational diabetes mellitus at term pregnancy: a systematic review and meta-analysis
Source: BMC Pregnancy Childbirth. 2023 Jul 12;23:509. doi: 10.1186/s12884-023-05779-z (PMC10339546; doi:10.1186/s12884-023-05779-z)
Supplement: Supplementary file 5 — Supplementary Material 5: Table S5 [file 12884_2023_5779_MOESM5_ESM.docx]

**Table S5.** Summary of findings table (GRADE)

| **Comparison of adverse maternal and perinatal outcomes between induction and expectant management among women with GDM at term pregnancy** | | | | | | |
| --- | --- | --- | --- | --- | --- | --- |
| **Patient or population:** Women with GDM at term pregnancy  **Intervention:** IOL  **Comparison:** Expectant Management | | | | | | |
| **Outcomes** | **№ of participants (studies) Follow-up** | **Certainty of the evidence (GRADE)** | **Relative effect (95% CI)** | **Anticipated absolute effects** | | |
|  |  |  |  | **Risk with EM** | | **Risk difference with IOL** |
| CS | 674 (3 RCTs) | ⨁⨁◯◯ Low^a,b,c,d,e^ | **OR 0.95** (0.64 to 1.43) | 179 per 1,000 | **7 fewer per 1,000** (56 fewer to 59 more) | |
|  | 12943 (8 observational studies) | ⨁◯◯◯ Very low^f,g^ | **OR 1.03** (0.79 to 1.34) | 193 per 1,000 | **5 more per 1,000** (34 fewer to 50 more) | |
| Macrosomia | 625 (2 RCTs) | ⨁⨁◯◯ Low^b,c,e^ | **OR 0.49** (0.30 to 0.81) | 164 per 1,000 | **76 fewer per 1,000** (108 fewer to 27 fewer) | |
|  | 11759 (6 observational studies) | ⨁◯◯◯ Very low^f,h^ | **OR 0.64** (0.54 to 0.77) | 115 per 1,000 | **38 fewer per 1,000** (49 fewer to 24 fewer) | |
| LGA | 200 (1 RCT) | ⨁⨁◯◯ Low^d,e,i^ | **OR 0.37** (0.17 to 0.83) | 230 per 1,000 | **130 fewer per 1,000** (182 fewer to 31 fewer) | |
|  | 11120 (3 observational studies) | ⨁◯◯◯ Very low^j^ | **OR 0.88** (0.72 to 1.06) | 117 per 1,000 | **13 fewer per 1,000** (30 fewer to 6 more) | |
| Instrumental vaginal delivery | 474 (2 RCTs) | ⨁⨁◯◯ Low^b,e^ | **OR 0.84** (0.47 to 1.50) | 119 per 1,000 | **17 fewer per 1,000** (59 fewer to 49 more) | |
|  | 8714 (3 observational studies) | ⨁⨁◯◯ Low^k,l^ | **OR 1.00** (0.84 to 1.18) | 114 per 1,000 | **0 fewer per 1,000** (16 fewer to 18 more) | |
| Severe perineal lacerations | 273 (1 RCT) | ⨁⨁◯◯ Low^b,e^ | **OR 0.13** (0.01 to 2.52) | 23 per 1,000 | **20 fewer per 1,000** (23 fewer to 33 more) | |
|  | 8556 (2 observational studies) | ⨁◯◯◯ Very low^e^ | **OR 0.59** (0.39 to 0.88) | 29 per 1,000 | **12 fewer per 1,000** (18 fewer to 3 fewer) | |
| ICU | 425 (1 RCT) | ⨁⨁◯◯ Low^b,e^ | **OR 1.49** (0.25 to 8.98) | 9 per 1,000 | **5 more per 1,000** (7 fewer to 70 more) | |
| Shoulder dystocia | 625 (2 RCTs) | ⨁⨁◯◯ Low^b,e^ | **OR 0.75** (0.04 to 15.49) | 13 per 1,000 | **3 fewer per 1,000** (12 fewer to 155 more) | |
|  | 11660 (5 observational studies) | ⨁◯◯◯ Very low^f,g^ | **OR 0.79** (0.48 to 1.30) | 23 per 1,000 | **5 fewer per 1,000** (12 fewer to 7 more) | |
| NICU | 425 (1 RCT) | ⨁⨁◯◯ Low^b,e^ | **OR 0.99** (0.14 to 7.06) | 9 per 1,000 | **0 fewer per 1,000** (8 fewer to 54 more) | |
|  | 8655 (3 observational studies) | ⨁◯◯◯ Very low^h,m^ | **OR 1.41** (0.85 to 2.32) | 96 per 1,000 | **34 more per 1,000** (13 fewer to 102 more) | |
| 5-min Apgar score <7 | 425 (1 RCT) | ⨁⨁◯◯ Low^b,e^ | **OR 4.98** (0.24 to 104.28) | 0 per 1,000 | **0 fewer per 1,000** (0 fewer to 0 fewer) | |
|  | 2518 (3 observational studies) | ⨁◯◯◯ Very low^e,n^ | **OR 0.40** (0.10 to 1.55) | 6 per 1,000 | **4 fewer per 1,000** (6 fewer to 3 more) | |
| Neonatal acidemia | 425 (1 RCT) | ⨁⨁◯◯ Low^b,e^ | **OR 7.00** (0.36 to 136.35) | 0 per 1,000 | **0 fewer per 1,000** (0 fewer to 0 fewer) | |
|  | 2139 (1 observational study) | ⨁◯◯◯ Very low^e^ | **OR 1.16** (0.24 to 5.48) | 5 per 1,000 | **1 more per 1,000** (3 fewer to 20 more) | |
| Perinatal mortality | 9195 (5 observational studies) | ⨁◯◯◯ Very low^e,l,o^ | **OR 0.64** (0.16 to 2.58) | 2 per 1,000 | **1 fewer per 1,000** (1 fewer to 3 more) | |
| ***The risk in the intervention group** (and its 95% confidence interval) is based on the assumed risk in the comparison group and the **relative effect** of the intervention (and its 95% CI).  **CI:** confidence interval; **OR:** odds ratio | | | | | | |
| **GRADE Working Group grades of evidence** **High certainty:** we are very confident that the true effect lies close to that of the estimate of the effect. **Moderate certainty:** we are moderately confident in the effect estimate: the true effect is likely to be close to the estimate of the effect, but there is a possibility that it is substantially different. **Low certainty:** our confidence in the effect estimate is limited: the true effect may be substantially different from the estimate of the effect. **Very low certainty:** we have very little confidence in the effect estimate: the true effect is likely to be substantially different from the estimate of effect. | | | | | | |

#### Explanations

a. 2 trials with unclear risk for most domains ( Kjos 1993, Singh 2013)

b. 1 trial (Alberico 2017) was open label, unable to blind women and healthcare professionals to the intervention, with high risk of performance and detection bias

c. 1 trial only included GDM women controlled by medical nutrition therapy (Singh 2013)

d. 1 trial only included insulin-treated GDM (Kjos 1993)

e. Relatively small sample size and a wide confidence interval

f. 3 studies had high risk of bias and no adjustment for the outcome, of which two had a historical comparison group (Lurie 1996, Conway 1998), and the other one with incomparable control group (Rayburn 2005);

g. Point effect estimate varied in direction while most of the studies included null value

h. 1 study with relatively small sample size (Alberico 2010)

i. The trial with unclear risk of bias for most of the domains (Kjos 1993)

j. 1 study (Conway 1998) had a historical comparison and no adjustment for the outcome (Conway 1998)

k. 1 study had high risk of bias, with a historical comparison and no adjustment for the outcome (Lurie 1996)

l. All of the studies included null value

m. Two studies showed a significantly protective effect (Melamed 2016, Vintner 2019)

n. 1 study had high risk of bias, with a historical comparison group and no adjustment for the outcome (Rayburn 2005)

o. 2 studies had high risk of bias, with a historical comparison group and no adjustment for the outcome (Lurie 1996, Rayburn 2005)
