# Supplementary material for: Comparison of adverse maternal and perinatal outcomes between induction and expectant management among women with gestational diabetes mellitus at term pregnancy: a systematic review and meta-analysis
Source: BMC Pregnancy Childbirth. 2023 Jul 12;23:509. doi: 10.1186/s12884-023-05779-z (PMC10339546; doi:10.1186/s12884-023-05779-z)
Supplement: Supplementary file 11 — Supplementary Material 11: Figure S7 [file 12884_2023_5779_MOESM11_ESM.docx]

**Fig. S7** Forest plot for NICU comparing induction with expectant management in women with GDM. Reference citations for studies can be found in Table 1. IOL, induction of labor; EM, expectant management
