# Supplementary material for: Comparison of adverse maternal and perinatal outcomes between induction and expectant management among women with gestational diabetes mellitus at term pregnancy: a systematic review and meta-analysis
Source: BMC Pregnancy Childbirth. 2023 Jul 12;23:509. doi: 10.1186/s12884-023-05779-z (PMC10339546; doi:10.1186/s12884-023-05779-z)
Supplement: Supplementary file 13 — Supplementary Material 13: Figure S9 [file 12884_2023_5779_MOESM13_ESM.docx]

**Fig. S9** Forest plot for neonatal acidemia comparing induction with expectant management in women with GDM. Reference citations for studies can be found in Table 1. One RCT (Alberico 2017) had no events recorded in expectant management group and a fixed correction using 0.5 to each cell of the 2X2 tables was applied for individual study odds ratio calculation. IOL, induction of labor
