# Supplementary material for: Comparison of adverse maternal and perinatal outcomes between induction and expectant management among women with gestational diabetes mellitus at term pregnancy: a systematic review and meta-analysis
Source: BMC Pregnancy Childbirth. 2023 Jul 12;23:509. doi: 10.1186/s12884-023-05779-z (PMC10339546; doi:10.1186/s12884-023-05779-z)
Supplement: Supplementary file 14 — Supplementary Material 14: Figure S10 [file 12884_2023_5779_MOESM14_ESM.docx]

**Fig. S10** Forest plot for perinatal mortality comparing induction with expectant management in women with GDM. Reference citations for studies can be found in Table 1. Four observational studies (Alberico 2010; Melamed 2016; Rayburn 2005; Vitner 2019) had no events recorded in induction group, and one study (Lurie 1996) had no events recorded in expectant management group. A fixed correction using 0.5 to each cell of the 2X2 tables was applied for individual study odds ratio calculation. IOL, induction of labor
