# Supplementary material for: Comparison of adverse maternal and perinatal outcomes between induction and expectant management among women with gestational diabetes mellitus at term pregnancy: a systematic review and meta-analysis
Source: BMC Pregnancy Childbirth. 2023 Jul 12;23:509. doi: 10.1186/s12884-023-05779-z (PMC10339546; doi:10.1186/s12884-023-05779-z)
Supplement: Supplementary file 15 — Supplementary Material 15: Figure S11 [file 12884_2023_5779_MOESM15_ESM.docx]

A) CS

B) Macrosomia

**Fig. S11** Funnel plot to assess publication bias for primary outcomes A) CS B) Macrosomia
