# Supplementary material for: Comparison of adverse maternal and perinatal outcomes between induction and expectant management among women with gestational diabetes mellitus at term pregnancy: a systematic review and meta-analysis
Source: BMC Pregnancy Childbirth. 2023 Jul 12;23:509. doi: 10.1186/s12884-023-05779-z (PMC10339546; doi:10.1186/s12884-023-05779-z)
Supplement: Supplementary file 17 — Supplementary Material 17: Table S4 [file 12884_2023_5779_MOESM17_ESM.docx]

**Table S4.** Pooled estimates for the effect of induction on primary outcomes in observational studies

| Observational study | Events/Total, n/N | | OR  (95% CI) | Sensitivity analysis | | |
| --- | --- | --- | --- | --- | --- | --- |
|  | IOL | EM |  | Q | I^2^ (%) | Pooled OR *  (95% CI) |
| **CS** | | | | | | |
| Alberico et al, 2010 | 9/48 | 11/51 | 0.84 (0.31, 2.25) | 23.1 | 74 | 1.04 (0.79, 1.37) |
| Conway et al, 1998 | 343/1337 | 266/1227 | 1.25 (1.04, 1.50) | 12.4 | 52 | 0.97 (0.74, 1.28) |
| Feghali et al, 2016 | 15/55 | 104/528 | 1.53 (0.81, 2.87) | 21.1 | 72 | 0.99 (0.75, 1.30) |
| Lurie et al, 1996 | 22/96 | 31/164 | 1.28 (0.69, 2.36) | 22.4 | 73 | 1.00 (0.76, 1.33) |
| Melamed et al, 2016 | 198/1188 | 1114/5229 | 0.74 (0.63, 0.87) | 4.69 | 0 | 1.19 (1.04, 1.38) |
| Rayburn et al, 2005 | 18/143 | 16/137 | 1.09 (0.53, 2.23) | 23.08 | 74 | 1.02 (0.77, 1.36) |
| Sutton et al, 2014 | 3/48 | 77/553 | 0.41 (0.12, 1.36) | 21.1 | 72 | 1.07 (0.82, 1.39) |
| Vitner et al, 2019 | 58/380 | 246/1759 | 1.11 (0.81, 1.51) | 22.4 | 73 | 1.01 (0.74, 1.39) |
| No exclusion | 666/3295 | 1865/9648 | 1.06 (0.96, 1.17) | 23.2 | 70 | 1.03 (0.79, 1.34) |
| **Macrosomia** | | | | | | |
| Alberico et al, 2010 | 6/48 | 11/51 | 0.52 (0.18, 1.54) | 5.5 | 27 | 0.63 (0.51, 0.78) |
| Conway et al, 1998 | 120/1337 | 147/1227 | 0.72 (0.56, 0.93) | 4.8 | 16 | 0.57 (0.43, 0.75) |
| Lurie et al, 1996 | 9/96 | 30/164 | 0.46 (0.21, 1.02) | 4.9 | 18 | 0.65 (0.53, 0.79) |
| Melamed et al, 2016 | 108/1188 | 666/5229 | 0.69 (0.55, 0.85) | 5.4 | 26 | 0.56 (0.40, 0.78) |
| Rayburn et al, 2005 | 6/143 | 11/137 | 0.50 (0.18, 1.40) | 5.4 | 26 | 0.63 (0.51, 0.78) |
| Vitner et al, 2019 | 9/380 | 119/1759 | 0.33 (0.17, 0.66) | 1.7 | 0 | 0.68 (0.58, 0.80) |
| No exclusion | 258/3192 | 984/8567 | 0.68 (0.59, 0.78) | 5.7 | 12 | 0.64 (0.54, 0.77) |

Sensitivity analysis was performed by leaving out one observational study;  ^*^ Pooled OR referred to the OR where a corresponding study was excluded at one time by a random-effects model with the Mantel-Haenszel method;
