# Supplementary material for: Comparison of adverse maternal and perinatal outcomes between induction and expectant management among women with gestational diabetes mellitus at term pregnancy: a systematic review and meta-analysis
Source: BMC Pregnancy Childbirth. 2023 Jul 12;23:509. doi: 10.1186/s12884-023-05779-z (PMC10339546; doi:10.1186/s12884-023-05779-z)
Supplement: Supplementary file 18 — Supplementary Material 18: Table S1 [file 12884_2023_5779_MOESM18_ESM.docx]

**Table S1.** Articles excluded after full text review and reasons for exclusion (n=77**)**

| **Articles** | **Exclusion reasons** |
| --- | --- |
| Olerich et al: Cesarean section rates by induction of labor indication in diabetic pregnancies | Inappropriate comparator |
| Vilchez et al: Pregnancy, Labor and Neonatal Outcomes and Optimal Timing for Induction of Labor in Gestational Diabetes | Inappropriate comparator |
| Rayburn et al: Effect of labor induction on cesarean rates in diabetic pregnancies | Inappropriate comparator |
| Hamel et al: Labor Induction and Cesarean Delivery Risk in Women with Gestational Diabetes Mellitus | Inappropriate comparator |
| Mantovani et al: Early diagnosis, active management and early induction of labour in pre-existing and gestational diabetes: Effects on pregnancy and delivery outcomes | Inappropriate comparator |
| Mirghani et al: A simplified management of diabetic pregnant women | Inappropriate intervention |
| Schwartz et al: Elective Delivery of Large Infants in Diabetic Pregnancies: Consequences and Implications | Inappropriate intervention |
| Sirota et al: Is Pharmacological Therapy during Induction of Labor in Gestational Diabetes Mellitus Patients Needed? | Inappropriate intervention |
| Maso et al: "GINEXMAL RCT: Induction of labour versus expectant management in gestational diabetes pregnancies". | Protocol |
| Bas-Lando et al: Elective induction of labor in women with gestational diabetes mellitus: an intervention that modifies the risk of cesarean section | Inappropriate comparator |
| Mashini et al: Indications for and timing of delivery in diabetic pregnancies | Inappropriate comparator |
| Kapustin et al: Time and mode of delivery in diabetic pregnancy: a review | Review |
| Vilchez et al: Labor and neonatal outcomes after term induction of labor in gestational diabetes | Inappropriate comparator |
| Hochberg et al: Perinatal outcome following induction of labor in patients with good glycemic controlled gestational diabetes: does timing matter? | Inappropriate comparator |
| Metcalfe et al: Timing of delivery in women with diabetes: A population-based study | Unrelated outcomes |
| Worda et al: Randomized controlled trial of induction at 38 weeks versus 40 weeks gestation on maternal and infant outcomes in women with insulin-controlled gestational diabetes | Inappropriate comparator |
| Wimberley et al: When a pregnant women is diabetic: intrapartal care | Review |
| Liner et al: Induction of labor in patients with diabetes mellitus | Correspondence |
| Thung et al: Fetal surveillance and timing of delivery in pregnancy complicated by diabetes mellitus | Review |
| Witkop et al: Active compared with expectant delivery management in women with gestational diabetes: a systematic review | Review |
| Leeman et al: Induction of labour at 37-38 weeks in women with large fetuses decreases the likelihood of shoulder dystocia; however, overall benefit of early-term delivery has not been demonstrated | Incorrect patient population |
| Sugiyama et al: Management of gestational diabetes mellitus | Review |
| Grabowska et al: Labour in women with gestational diabetes mellitus | Inappropriate comparator |
| Zara et al: Medical and elective induction: A prospective randomized study | Incorrect patient population |
| Nicholson et al: Therapeutic management, delivery, and postpartum risk assessment and screening in gestational diabetes | Review |
| Herranz et al: Elective delivery in women with gestational diabetes mellitus | Review |
| Karmon et al: Decreased perinatal mortality among women with diet-controlled gestational diabetes mellitus | Inappropriate intervention |
| Crippa et al: Outcome of induction of labour in women with gestational diabetes and comparison with general obstetric population | Inappropriate comparator |
| Yogev et al: Active induction management of labor for diabetic pregnancies at term; mode of delivery and fetal outcome--a single center experience | Inappropriate comparator |
| Jiang et al: Effects of gestational diabetes mellitus on time to delivery and pregnancy outcomes in full-term pregnancies with dinoprostone labor induction | Inappropriate intervention |
| Atad et al: Induction of labor in gestational diabetes | Review |
| Boulvain et al: Elective delivery in diabetic pregnant women | Review |
| Arnold et al: Induction of labour in pregnant diabetics using vaginal prostaglandin E2 pessaries | Inappropriate intervention |
| Gawlik et al: Timing of elective repeat caesarean does matter: Importance of avoiding early-term delivery especially in diabetic patients | Inappropriate intervention |
| Duryea et al: The effect of expectant management of mild gestational diabetes at term on perinatal morbidities | Inappropriate comparator |
| Fraser et al: Gestational diabetes: After the ACHOIS trial | Review |
| Wagner et al: Association between time of delivery and composite adverse outcomes in pregnancies complicated by diabetes | Inappropriate intervention |
| Schwarz et al: Timing of delivery in the pregnant diabetic patient | Unrelated outcomes |
| Gabbe et al: General obstetric management of the diabetic pregnancy | Review |
| Khonjandi et al: Gestational diabetes: the dilemma of delivery | Inappropriate intervention |
| Huang et al: Intrapartum Results on Differing Degrees of Ketonuria in Nulliparous Women with Gestational Diabetes Mellitus during Spontaneous Labor | Inappropriate intervention |
| Dude et al: Association between Sonographic Estimated Fetal Weight and the Risk of Cesarean Delivery among Nulliparous Women with Diabetes in Pregnancy | Inappropriate intervention |
| Berger et al: Timing of delivery in women with diabetes in pregnancy | Review |
| Biesty et al: Planned birth at or near term for improving health outcomes for pregnant women with gestational diabetes and their infants | Review |
| Garabedian et al: Delivery (timing, route, peripartum glycemic control) in women with gestational diabetes mellitus | Review |
| Biesty et al: Planned birth at or near term for improving health outcomes for pregnant women with gestational diabetes and their infants | Review |
| Korkmazer et al: Gestational Diabetes: Screening, Management, Timing of Delivery | Review |
| Arnold et al: Induction of labor in pregnant diabetics using vaginal prostaglandin-E2 pessaries | Inappropriate intervention |
| Levy et al: Effect of labor induction on cesarean section rates in diabetic pregnancies | Inappropriate comparator |
| Hod et al: Antepartum management protocol: Timing and mode of delivery in gestational diabetes | Inappropriate intervention |
| Egan et al: A Review of the Pathophysiology and Management of Diabetes in Pregnancy | Review |
| Landon et al: Fetal surveillance and timing of delivery in pregnancy complicated by diabetes mellitus | Review |
| Boulvain et al: Elective delivery in diabetic pregnant women | Review |
| Sacks et al: Induction of labor versus conservative management of pregnant diabetic women | Review |
| Visser et al : Management of diabetes in pregnancy: antenatal follow-up and decisions concerning timing and mode of delivery | Review |
| Hersh et al: Induction of labor at 39 weeks of gestation versus expectant management for low-risk nulliparous women: a cost-effectiveness analysis | Incorrect patient population |
| Henry et al: Randomized trial of elective induction vs expectant management in diabetics | Conference abstract |
| Nayeri et al: Labor induction at 38 weeks versus expectant management of insulin-requiring diabetics in pregnancy: a cost effective analysis | Conference abstract |
| No name: Gestational Diabetes: induction Versus Expectant Management of Labour | Protocol |
| Alberico et al: Fulfilling professional responsibilities when counselling patients about Zika infection | Commentary |
| No name: Insulin Dependent Gestational Diabetes Mellitus: randomized Trial of Induction of Labour at 38 and 40 Weeks of Gestation | Protocol |
| No name: The Best Timing of Delivery in Women With GDM Study | Protocol |
| Niu et al: Timing of induction of labor among women with gestational diabetes (GDM) | Conference abstract |
| Rosenstein et al: The risk of stillbirth and infant death stratified by gestational age in women with gestational diabetes | Inappropriate intervention |
| Nau et al: Induction Compared to Expectant Management for Women With Gestational Diabetes at Term | Conference abstract |
| McElwee et al: The risk of perinatal mortality by week of expectant management in pregnancies complicated by diabetes | Conference abstract |
| No name: To understand whether in women diagnosed with gestational diabetes well controlled on diet therapy at term (39-40 gestational weeks) would be better to induce labour or expect the spontaneous onset of labour | Protocol |
| Cheng et al: Women with gestational diabetes mellitus: induction or spontaneous labor? | Conference abstract |
| Niu et al: What is the optimal gestational age for women with gestational diabetes type A1 to deliver? | Inappropriate intervention |
| Boulvain et al: Elective delivery in diabetic pregnant women | Review |
| Harper et al: Gestational Age of Delivery in Pregnancies Complicated by Diabetes | Incorrect patient population |
| Leaphart et al: Labor induction with a prenatal diagnosis of fetal macrosomia | Incorrect patient population |
| Peled et al: Gestational diabetes mellitus--implications of different treatment protocols | Inappropriate comparator |
| Hod et al: Antepartum management protocol. Timing and mode of delivery in gestational diabetes | Inappropriate comparator |
| Lurie et al: Outcome of pregnancy in class A1 and A2 gestational diabetic patients delivered beyond 40 weeks' gestation | Inappropriate intervention |
| Lyons et al: Timing of Delivery in Women with Diabetes: A Population-Based Study | Commentary |
| Alberico et al: Immediate delivery or expectant management in gestational diabetes at term: the GINEXMAL randomised controlled trial | Duplicate |
